# Accommodative behaviour and retinal defocus of children during prolonged viewing of electronic devices while wearing dual‐focus myopia control soft contact lenses

**DOI:** 10.1111/opo.13533

**Published:** 2025-05-28

**Authors:** Neeraj K. Singh, Pete Kollbaum

**Affiliations:** ^1^ School of Optometry Indiana University Bloomington Indiana USA

**Keywords:** accommodation, contact lenses, dual focus, myopia, retinal defocus

## Abstract

**Purpose:**

Chronic hyperopic defocus from inadequate accommodation during near tasks may be associated with axial eye growth. This study examined accommodative behaviour and retinal defocus in myopic and emmetropic children after 1 h of continuous electronic device use.

**Methods:**

Thirty‐four children (17 per group, aged 7–17) participated, including myopes wearing dual‐focus (DF) myopia control contact lenses (MiSight 1‐day) and uncorrected emmetropes. In phase one, on‐axis aberrometry data were collected at distances from 4.00 to 0.20 m, corresponding to target vergences from −0.25 to −5.00 D. In phase two, measurements were taken every 10 mins, as children watched a movie at 0.20 m for 60 min. Local refractive state measures were calculated from aberrometry measures and pooled within pupil areas corresponding to DF lens central and treatment zones. Retinal defocus was calculated by subtracting target vergence from the measured refractive state. Linear mixed‐effects models analysed group, target vergence and time effects on defocus, adjusting for pupil diameter.

**Results:**

Myopes with DF lenses showed greater hyperopic defocus (+0.58 D, *p* = 0.001) than uncorrected emmetropes during on‐axis viewing. Hyperopic defocus increased significantly over time in both groups with near viewing (*p* < 0.001). Myopic defocus induced by DF lenses was still present but decreased following 50 and 60 min of near viewing (*p* = 0.05 and *p* = 0.007, respectively).

**Conclusions:**

Periods of sustained near viewing of up to 1 h increased hyperopic defocus in both groups. However, DF contact lenses still introduced myopic defocus in myopic subjects throughout this time, supporting their potential to slow axial eye growth during periods of sustained near viewing. Further work will be helpful to understand how the sustained near viewing model and associated results of the current work relate to the real‐world environment, which may include potential breaks and/or longer total near viewing durations.

**Key points**

- Both myopic and emmetropic children exhibited a significant increase in hyperopic defocus over 60 min of sustained near work.
- Dual‐focus contact lenses maintained myopic defocus in myopic children throughout the viewing period.
- The findings highlight the importance of considering individual accommodative behaviours and visual habits when developing myopia control strategies, as prolonged near work can influence defocus dynamics over time.

## INTRODUCTION

The prevalence of myopia has reached epidemic proportions in certain populations, with rapid increases documented in regions such as East Asia, where rates exceed 80–90% in some age groups (e.g., 14–42 years).^1^, ^2^ Myopia, characterised by excessive eye elongation due to a disparity between the eye's optical power and axial length, is influenced by various factors besides genetics.^3^ Near work and other behavioural factors are associated with the onset and progression of myopia.^4^, ^5^, ^6^ Prolonged near tasks, such as schoolwork, homework, video games and mobile phone use, have been linked with an increased prevalence of myopia in children.^2^, ^6^, ^7^, ^8^, ^9^ It is hypothesised that inadequate accommodation (accommodative lag) during prolonged near work may introduce hyperopic defocus, thereby stimulating abnormal eye growth.^10^, ^11^, ^12^, ^13^, ^14^, ^15^

The prevalence of myopia in children has surged, with excessive electronic device use being a significant contributing factor, particularly following the COVID‐19 pandemic.^16^, ^17^ Even prior to the pandemic, mobile device usage among children was common, with daily usage averaging 2 h per person, a number that has since doubled across most age groups.^18^ In the United States, digital device use begins as early as infancy, with most children owning tablets by age 4 years.^19^, ^20^, ^21^ The COVID‐19 pandemic further increased device usage in children, especially in those aged 2–11 years.^16^, ^22^

Children's interaction with devices, particularly at close distances (10–25 cm), has been linked with myopia progression.^23^ Studies have shown that children often work at reduced distances during near tasks, which, when combined with accommodative lags, can expose them to hyperopic defocus, which is a risk factor for developing myopia.^9^, ^24^ Shorter viewing distances, such as those typical during smartphone use, can exacerbate the focusing demand, particularly in myopic children.^25^

Cross‐sectional studies have reported that children spending more than 2–4 h on devices are significantly more likely to experience myopia progression.^5^, ^6^ This increased screen time also reduces outdoor activities, a factor reported to be protective against myopia development.^6^, ^26^ Given these concerns, this study explores the accommodative behaviour and retinal defocus in children using electronic devices for up to 1 h of continuous use. It also examines differences in these responses between myopic children wearing dual‐focus (DF) myopia control contact lenses (CLs) and emmetropic children without any refractive correction.

## METHODS

### Participants

Thirty‐four myopic (−0.50 to −6.50 D) and emmetropic (−0.25 to +0.75 D) children (7–17 years of age) were recruited by word of mouth, from recruitment databases and using an advertisement circulated via email and social media platforms. Assuming an equal allocation of emmetropic and myopic children (i.e., *n*_*E*_ = *n*_*M*_ = 17, obtained with G*Power software V3.1.9.6; psychologie.hhu.de), this sample size was expected to enable detection of a standardised effect size of ~1 for the difference between two independent means with 80% power and a two‐sided Type I error rate of 0.05.

Children were screened with a routine eye examination to determine monocular distance subjective refraction, visual acuity and general ocular health. Myopia was defined as spherical equivalent refraction of ≤− 0.50 D.^27^ Children with best‐corrected visual acuity of 0.00 logMAR (6/6) or better, no manifest strabismus or amblyopia and no history of refractive surgery or ocular disease were recruited. The study protocol followed the tenets of the Declaration of Helsinki, and approval was obtained from the Indiana University research ethics committee. Written informed consent and/or assent was obtained from each child and their parents before participating in the study.

### Measurement procedure

Wavefront data were recorded from the right eye of emmetropic children not wearing any correction and the right eye of myopic children wearing best‐corrected dual‐focus (DF) CLs (MiSight; coopervision.com) in both eyes. Wavefront measures were acquired using a previously validated^28^, ^29^ high‐resolution pyramidal wavefront sensing aberrometer (Osiris, Costruzione Strumenti Oftalmici, csoitalia.it). The aberrometer captures the combined wavefront error of the eye and DF lens in situ, reflecting the lens's optical design; these data were subsequently analysed to derive zone‐specific refractive states. The study was conducted in two phases at the same measurement session. In the first phase, the baseline accommodative response of each child was established. Children viewed high‐contrast stimuli binocularly, with the target positioned at 4.00, 1.00, 0.50, 0.33, 0.25 and 0.20 m (corresponding to target vergences of −0.25, −1.00, −2.00, −3.00, −4.00, −5.00 D) in front of the right eye. All measurements were acquired from the right eye which remained in approximately the same axial position relative to the targets while the unmeasured left eye converged to fixate the target. Eye stability during the procedure was maintained by a chin and forehead rest. On‐axis (foveal) measurements were achieved by aligning the fixation target with the instrument measurement axis. The measurement axis of the instrument was aligned with the primary line of sight of the right eye of each participant before performing any measurements. An infrared beam splitter introduced into the measurement path allowed open‐field binocular viewing. For this phase of the experiment, targets consisted of randomised high‐contrast letters (0.30 logMAR [6/12], scaled for each viewing distance) presented on an iPhone 6 s (apple.com) with screen luminance of 148 ± 0.38 cd/m^2^ while the room illumination was approximately 5 lux. For distance viewing, participants observed the 0.30 logMAR (6/12) line on an illuminated Early Treatment of Diabetic Retinopathy Study chart at 4.00 m. Three measurements were acquired at each target vergence.

In the second phase of the experiment, participants engaged in a ‘sustained’ near viewing task for 60 min at 20 cm (−5.00 D target vergence). During this task, children watched a movie (‘The Lion King’) on an Apple iPhone 6 s (apple.com), with the screen brightness set to its lowest setting (screen luminance mean ± SD: 2.63 ± 0.30 cd/m^2^) to maximise pupil diameter in a low illumination room environment (5.44 ± 0.22 lux). Four repeated wavefront aberrometry measures were recorded every 10 min over the 60‐min period.

To maximise pupil diameter and create a naturalistic viewing environment (i.e., participants being naïve to the timing of measurement acquisition), the experimental set‐up was enclosed with black curtains. The low illumination setting was also chosen to reveal the effect of tonic accommodation^30^ and to mimic a common real‐life scenario of electronic usage under dim lighting conditions,^31^ such as at bedtime.^31^, ^32^ At the beginning of the near task session, children were instructed to watch the movie as they would naturally, without any interruptions or additional instructions throughout the session, to preserve the naturalistic viewing scenario. The children were also advised to ignore any movements or noise generated by the examiner during the procedure and to maximise their focus on the viewing task to the largest extent possible.

### Contact lenses

The DF CL (MiSight 1‐day, omafilcon A, CooperVision, Inc., coopervision.com) utilised to correct myopic subjects during testing had a base curve of 8.70 mm and a diameter of 14.20 mm. Myopic eyes were bilaterally fitted with the soft DF CLs to optimise monocular distance vision as determined during the eye examination (maximum plus or minimum minus correction). The DF CLs consisted of four alternating distance and near zones from the innermost to the outermost rings.^12^, ^33^, ^34^

### Data analysis

**FIGURE 1:**
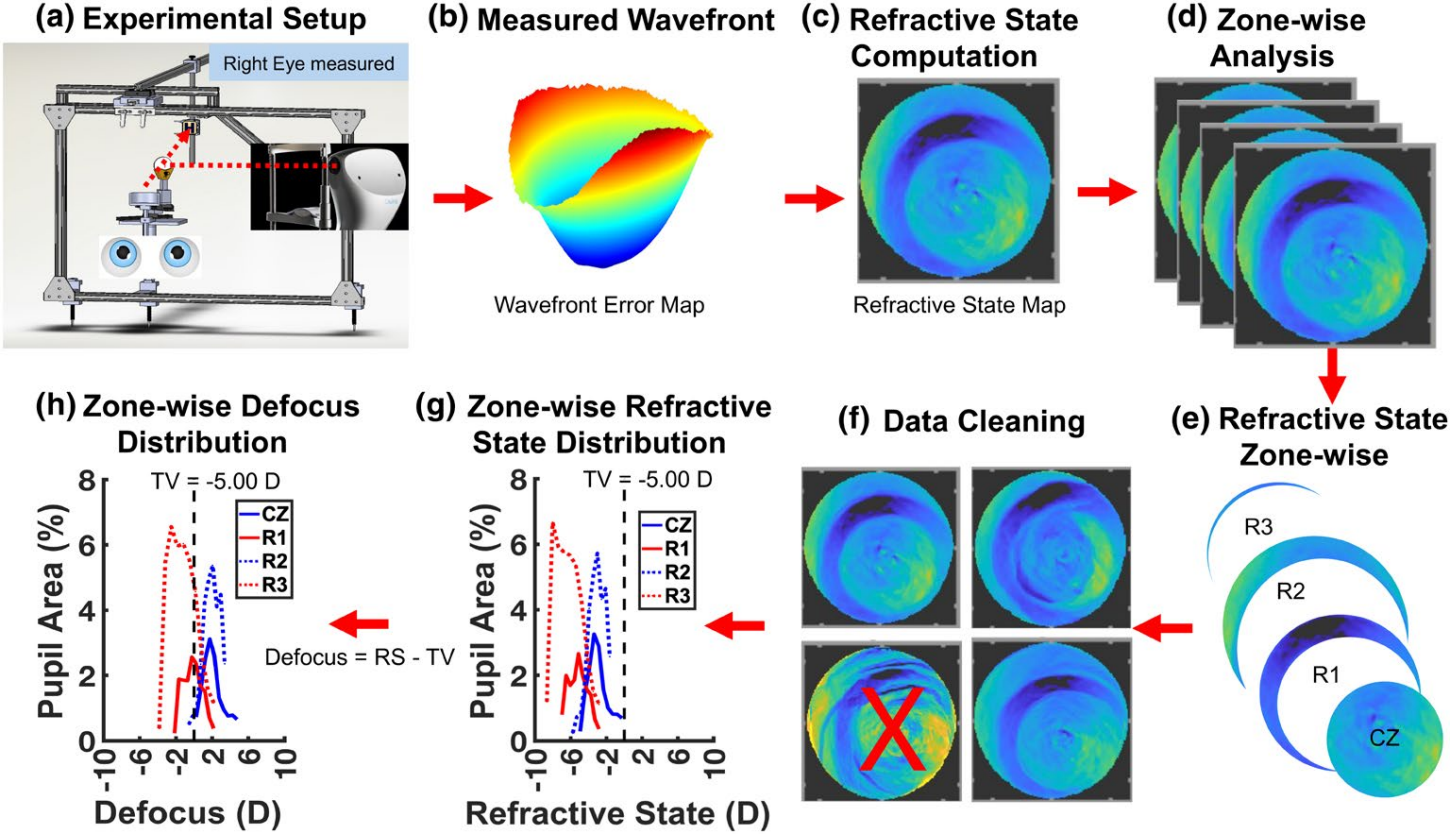
The experimental set‐up (a) and the order of data collection and processing (b–h). The wavefront (b) was captured using a Pyramidal wavefront sensing aberrometer on a sample of a myopic eye wearing a dual‐focus contact lens. Three repeated measurements were acquired while subjects viewed a letter stimulus at different distances (on‐axis condition only). During the near viewing task, four repeated measurements were acquired every 10 min while children viewed a movie at 20 cm (target vergence [TV] = −5.00 D) for 60 min. Using custom MATLAB software, the local refractive state was calculated for each repeat (c). Zone‐wise analysis was performed to identify the zone boundaries manually (d) and extract the local refractive state of each zone for each repeated measurement (e). Refractive state data of each repeated measurement were corrected for prism and errors due to lens decentration. Data sets were examined manually (f) to exclude instances of corruption caused by links, eyelashes, tear breakup, temporary interruptions in gaze or accommodation, as well as any outliers. The refractive state data for each segmented zone (central zone [CZ], as well as the first [R1], second [R2] and third [R3] treatment zones) were extracted, and a plot of refractive state distribution across each zone was created (g). The target vergence (TV) was subtracted to compute and plot the defocus distribution for each zone (h). RS, refractive state.

Wavefront data for the full natural pupil were exported and processed using customised software (Indiana Wavefront Analyser [IWA]) implemented in MATLAB (mathworks.com). Local integration of the slope data provided zonally reconstructed wavefronts avoiding Zernike fitting.^35^ The radial slope divided by the distance from the pupil centre (uncorrected emmetropic eyes) or CL (myopic eyes wearing DF CL) centre yielded the wavefront vergence at each point in the pupil.^36^ Refractive state map coordinates were aligned with the CL centre using a zone‐wise analysis approach as reported previously.^12^ The average refractive state within each of the central and annular treatment zones for each eye was quantified as the sum of all points divided by the number of points (Figure 1). The positioning of lenses relative to the pupil centre of the DF zonal CLs was determined by identifying the boundary between the central and first annular zones, and subsequently between the first, second and third annular zones, based on the refractive state maps and known lens ring geometry. While CL decentration was considered in myopic eyes wearing MiSight CLs, this was not applicable for the emmetropic eyes, as no optical correction was worn. For the uncorrected emmetropic eyes, equivalent geographic regions within the pupil were computed, assuming a CL centred on the pupil and using the standard MiSight 1‐day DF CL design ring dimensions. Repeated measurements were manually reviewed and up to three visually acceptable measurements were selected for each time point and measurement condition for each eye. Data sets excluded any instances of corruption due to blinks, eyelashes, tear breakup or temporary interruptions in gaze and accommodation. The refractive state data for each repeated measurement were corrected for prism errors and lens decentration. In each distance‐corrected eye, differences between the refractive state and the target vergence yielded the retinal image defocus due to accommodative error (accommodative error = refractive state − target vergence). Accommodative lead (myopic defocus) and lag (hyperopic defocus) were represented using negative and positive signs, respectively. Kernel density plots across the full pupil were used to quantify the proportion of hyperopically, myopically and focused light passing through the lens and pupil, using clinical refractive state thresholds to define myopia (≤−0.50) and hyperopia (≥+ 0.75 D).^27^, ^37^ Across conditions/eyes, all refractive state or defocus data were pooled for every point within each visible lens zone and within the pupil regardless of pupil diameter.

The baseline characteristics of the two groups were compared using an independent samples Mann–Whitney *U*‐test for age and Fisher's exact test for sex to ensure that the groups were balanced with respect to demographic characteristics. For descriptive purposes, sample means (95% CI) were calculated. For inference, linear mixed effects (LME) regression was used to analyse accommodative error (defocus) data from the two experimental phases. Recall that phase 1 involved viewing targets at different distances (on‐axis condition), while the second phase involved participants viewing electronic devices continuously at a fixed distance of 20 cm for 60 min. In both phases, repeated measurements were made on the same children within each group: emmetropes and myopes. For each task, a separate LME regression model was fitted to the data for the central zone (CZ) and the first treatment zone (R1). In each model, mean defocus (i.e., within‐subject average of up to three repeated measurements at each target vergence or time point) was used as the response measure. The fixed‐effect predictors were group (dummy coded with emmetropes as the reference class), target vergence or time and (group × time) interaction or (group × target vergence) interaction (on‐axis condition). Each model was also statistically adjusted by pupil diameter. A separate LME model was fitted with pupil diameter as the response variable, with group and target vergence as fixed predictors. To account for inter‐subject variability and correlated residuals due to repeated measures within subjects, all models included random effects representing subject‐specific intercepts and slopes. Models were fitted using restricted maximum likelihood estimation with the ‘lmer’ function from the ‘lmerTest’ package in R (Version 4.4.1, r‐project.org). Fixed effects were tested using ANOVA with Satterthwaite degrees of freedom.^38^

While treating time as a continuous variable provided insights into the overall trend of defocus over the entire period, further analysis was performed to examine changes in defocus over specific time points. Therefore, an analysis treating time as a categorical variable was performed and another LME model fitted. This approach allowed for the examination of differences in introduced defocus at specific time points, providing more detailed insights into how defocus changes over distinct periods. Post hoc comparisons were performed on significant fixed effects using Dunnett's method, which adjusts *p*‐values to control the family‐wise error rate, comparing each time point to the baseline.

## RESULTS

The mean ± SD age of the emmetropic and myopic children was 12 ± 3.50 years (range: 7–17 years, 11 boys) and 14 ± 2.34 years (range: 10–17 years, 9 boys), respectively. A Mann–Whitney *U*‐test revealed no significant difference in age between the emmetropic and myopic groups (*p* = 0.23). Additionally, Fisher's exact test indicated no significant difference in sex distribution between the two groups (*p* = 0.73). The mean spherical equivalent refraction for the myopic and emmetropic children was −2.00 ± 1.61 D and +0.29 ± 0.44 D, respectively.

**FIGURE 2:**
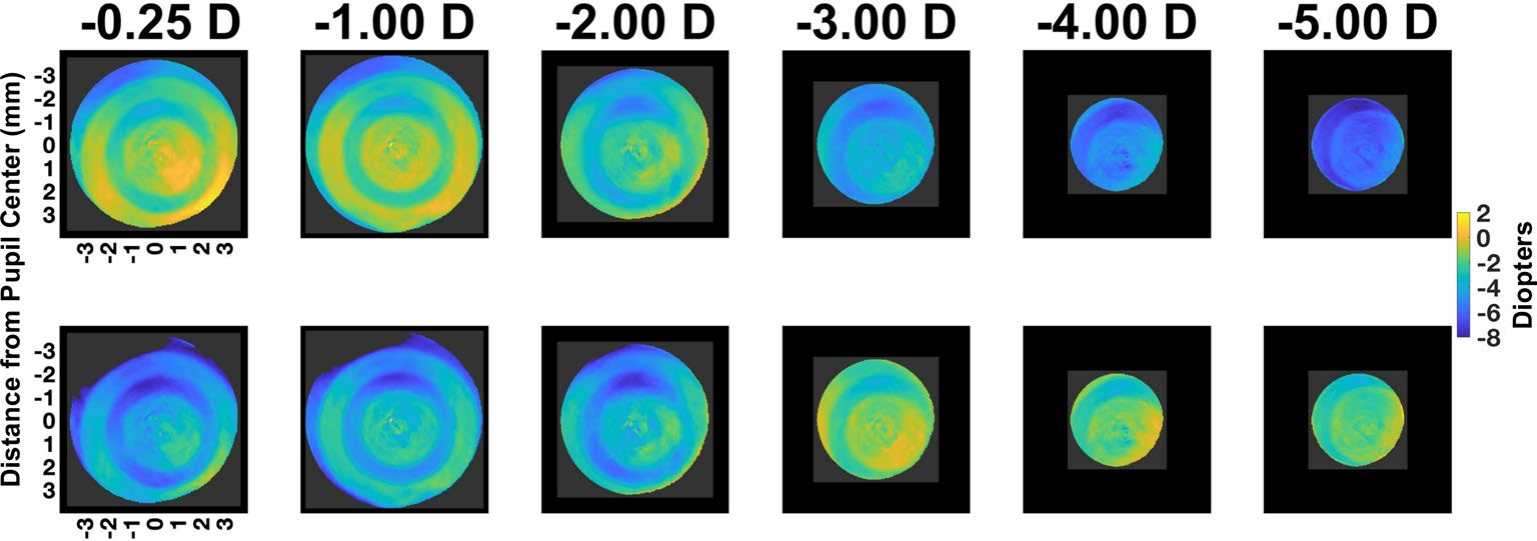
Coloured maps of the refractive state (top panels) and defocus (refractive state—target vergence [TV]; bottom panels) from one sample eye wearing dual‐focus contact lens at different target vergences. The on‐axis foveal measurements ranged from −0.25 to −5.00 D target vergences. As the target vergences increased, the pupil size decreased and the reported map contains smaller pupil data. However, since the contact lenses have fixed size, the central zone of contact lenses covers a larger proportion of the pupil.

Sample refractive state maps (Figure 2, top panels) and defocus maps (Figure 2, bottom panels) are provided for a 14‐year‐old myopic child with a spherical equivalent of −0.50 D, who viewed the letter target at each viewing distance (4.00, 1.00, 0.50, 0.33, 0.25 and 0.20 m) (Figure 2, columns left to right). These sample maps illustrate each zone of the DF CL, from the inner to the outer lens regions across different target vergences. In the top panels, a gradual transition in the refractive state within the central distance zone is observed, progressing from yellow through green to blue as the target vergence increased (viewing distance decreased, left to right). The defocus maps (Figure 2, bottom panels) reveal a similar gradual transition of the central distance ring to yellow, reflecting the on‐eye combined effects of lens geometry and accommodative error on retinal image defocus. Note that due to pupil miosis at the nearest target vergences in this sample participant, the central zones of the CL appear slightly larger, as they cover a greater proportion of the smaller pupil.

**FIGURE 3:**
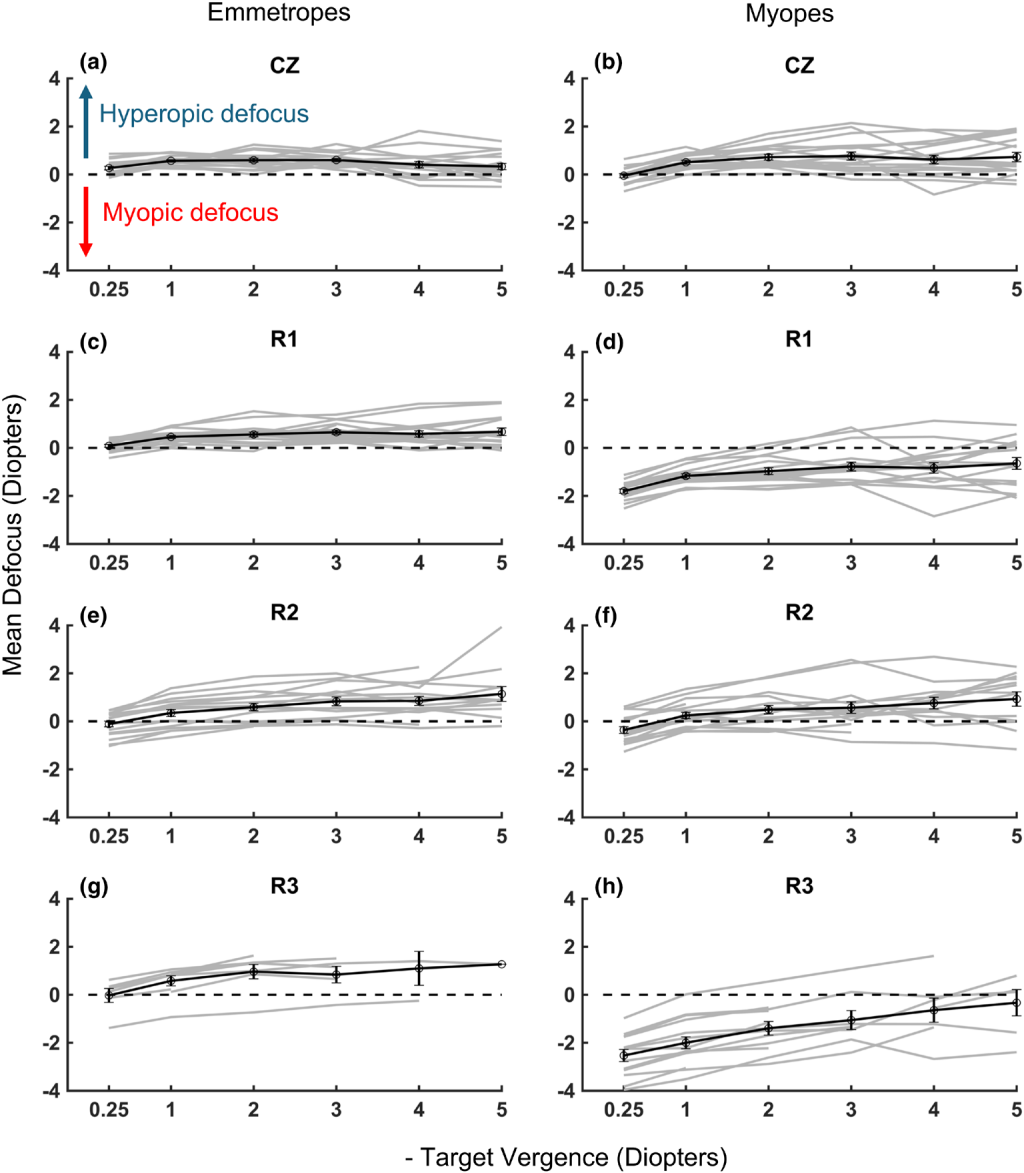
Mean retinal defocus (defined as refractive state minus target vergence, in dioptres) plotted against target vergence (in dioptres) for all participants. Data are shown for each optical zone—central zone (CZ), first ring (R1), second ring (R2) and third ring (R3). Panels (a, c, e, g) display simulated zone‐wise analysis for emmetropic children, assuming a centred dual‐focus ring configuration in the absence of corrective lenses. Panels (b, d, f, h) illustrate data for myopic children wearing dual‐focus contact lenses. For both groups (17 children each), the mean defocus is represented by a solid black line with symbols, while individual subject data are shown as grey lines. Error bars represent ±1 standard error of the mean.

Refractive state (RS) minus target vergence (TV) yields retinal defocus (defocus = RS − TV; e.g., −4.50 D – (−5.00 D) = +0.50 D, indicating hyperopic defocus of +0.50 D for a target at 20 cm).^12^ The average defocus over the range of viewing distances for both myopic and emmetropic children is plotted in Figure 3. Both myopic children wearing DF CLs and uncorrected emmetropic children accommodated well with minimal lag. Small errors in accommodation in emmetropic eyes yielded +0.46 ± 0.13 D defocus across all TVs. Although the errors were larger in the DF‐corrected myopic group (+0.57 ± 0.32 D), these small errors are not clinically significant.^39^ Analysis using an LME model, with target vergence as a continuous predictor, revealed significant between‐subject variability (standard deviation = 0.32), underscoring the impact of individual differences on defocus measurements. The fixed effect of TV was not significant (*p* = 0.56), indicating that defocus was not notably influenced by changes in TV alone. However, pupil size had a significant effect on defocus (*p* < 0.001), showing that variations in pupil diameter contributed to defocus values across measurements. The group effect was also significant (estimate = 0.58, *p* = 0.001), with myopes demonstrating an average defocus that was +0.58 D higher than that of emmetropes. Furthermore, a significant interaction between group and TV (estimate = 0.20, *p* = 0.002) revealed that, with each dioptre increase in target vergence, defocus increased by an additional +0.20 D in myopes, compared with emmetropes. This finding suggests that myopes experienced a greater increase in hyperopic defocus with increasing TV, indicating differential defocus behaviour between refractive groups under varying vergence demands.

**FIGURE 4:**
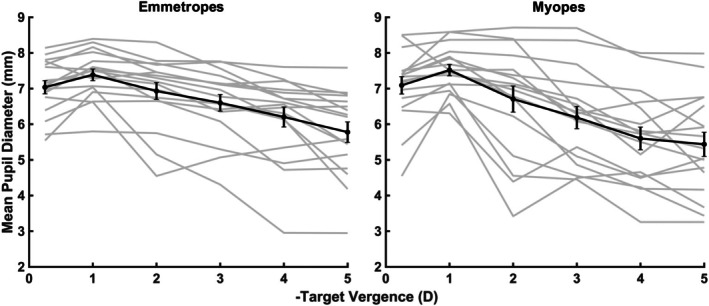
Mean pupil diameter as a function of target vergence (dioptres). The mean defocus of 17 children in each emmetropic (left panel) and myopic group (right panel) is plotted in black solid line and symbols, while each individual children's data are plotted in grey lines. Error bars are ±1 standard error of the mean.

ANOVA revealed no significant difference in pupil size between the emmetropic and myopic groups (*p* = 0.54, Figure 4). However, a significant main effect of TV on pupil size was found (*p* = 0.009), signifying that pupil size varies substantially with changes in vergence, as expected. The interaction between group and TV was not significant (*p* = 0.89), suggesting that the effect of TV on pupil size was uniform across both emmetropic and myopic subjects.

**FIGURE 5:**
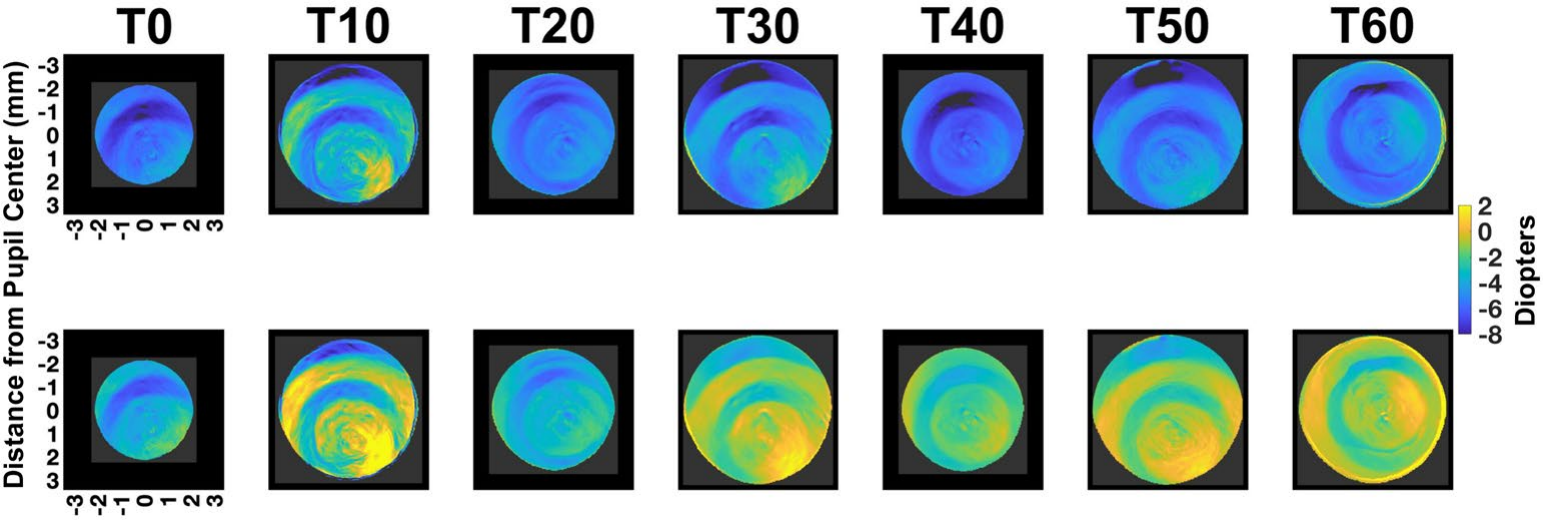
Refractive state (top panels) and defocus maps (calculated as refractive state minus target vergence; bottom panels) for a sample child (the same subject as in Figure 2) wearing dual‐focus contact lenses while viewing a movie on a mobile screen at a distance of 20 cm for 60 min. Measurements were taken at 10‐min intervals. The DF CLs display four concentric zones, progressing from the innermost to the outermost: two correction zones (centre zone [CZ] and R2) and two treatment zones (R1 and R3) across all time points. Defocus is calculated as the difference between refractive state (RS) and target vergence (TV), with positive defocus values indicating hyperopic defocus. Maps represent the full natural pupil.

The data for the same child shown in Figure 2 when viewing a mobile device for 60 min at 20 cm (target vergence = −5.00 D) is presented in Figure 5. The top panels show the refractive state maps (top panels) and defocus maps (bottom panels) at different time points (0–60 min, left to right). Note, the bottom is merely a mathematical subtraction of the TV. Specifically, the defocus maps were derived after subtracting the −5.00 D target vergence from the refractive state to show the combined effect of lens geometry and accommodation on retinal image defocus.

**FIGURE 6:**
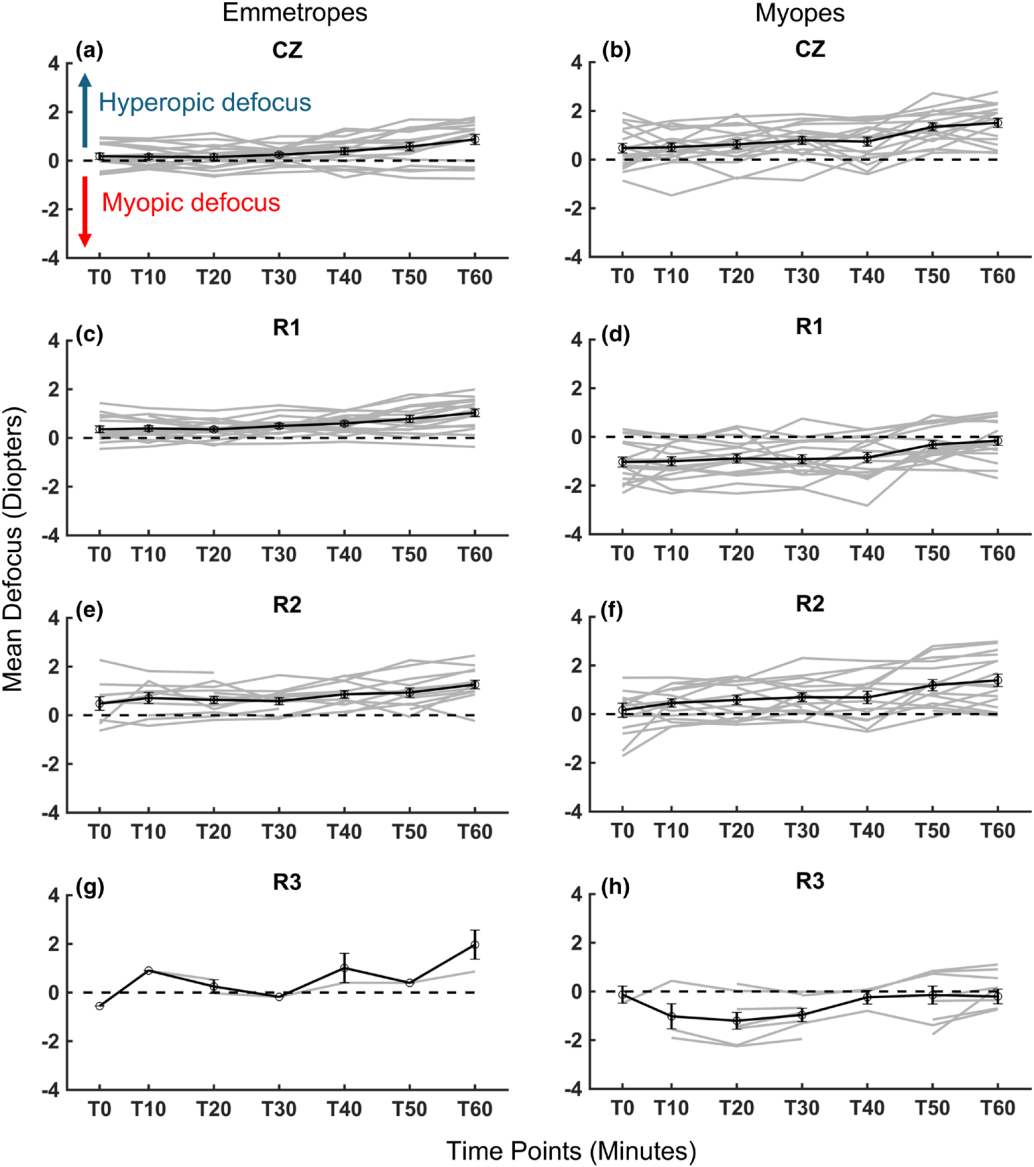
Mean defocus (Refractive state – target vergence, in dioptres) over time for all children. T0–T60 indicates the elapsed time in minutes. Myopic children wearing dual‐focus (DF) contact lenses are shown in panels (b, d, f, h). Data for emmetropic children (a, c, e, g) are based on a simulated zone‐wise analysis using the DF ring dimensions. Each group's mean defocus is represented by black solid lines and symbols, with individual data points for each child plotted in grey lines. Due to pupil miosis at −5.00 D target vergence, individual eye data for R3 (g, h) are not available. Error bars denote ±1 standard error of the mean. CZ, central zone; R1, R2 and R3 refer to the first, second and third treatment zones, respectively.

Examining the results of all subjects during sustained screen exposure at a 20‐cm viewing distance, both emmetropic and myopic children exhibited progressive shifts in their accommodative responses over time (Figure 6). Myopic children demonstrated an increase in hyperopic defocus, beginning at a higher baseline (T0) level of +0.47 ± 0.84 D and rising to +1.51 ± 0.80 D by 60 min (T60). This trend also persisted in the second correction zone (R2). Specifically, for myopic children wearing DF CLs, the average hyperopic defocus within R2 reached +1.19 ± 0.70 D at 50 min (T50) and +1.39 ± 0.80 D at T60. In the DF lens treatment ring (R1), myopic children initially experienced an average of −1.00 ± 0.84 D of myopic defocus at T0, but this effect diminished significantly to −0.32 ± 0.70 (*p* = 0.002) at T50 and −0.16 ± 0.80 (*p* < 0.001) at T60. In emmetropic children, hyperopic defocus in the equivalent central correction zone (CZ) gradually increased from an initial +0.18 ± 0.58 D at baseline (T0) to +0.87 ± 0.85 D at T60, and this trend persisted in the R2‐equivalent treatment zone. For emmetropes, the R1‐equivalent zone showed a progressive increase in hyperopic defocus, rising from +0.36 ± 0.56 D at T0 to +1.03 ± 00.65 D at T60. These findings illustrate the cumulative effect of sustained near work on hyperopic defocus in both refractive groups.

Across this duration, substantial individual variability in accommodative responses was observed, indicated by a large between‐subject variance (standard deviation = 0.62), highlighting diverse responses to visual demands among the child participants. No significant effect of refractive group (emmetropic vs. myopic) on average defocus levels over time (*p* = 0.21) was observed, suggesting that both groups displayed similar overall defocus. Additionally, the group‐by‐time interaction was not significant (*p* = 0.38), indicating that the increase in the rate of defocus over time was comparable between emmetropic and myopic participants. A significant main effect of time (*p* = 0.003) was observed, revealing the progressive increase in hyperopic defocus throughout the 60‐min session described across both groups. Post hoc analysis using Dunnett's test confirmed the time‐dependent progression of defocus. Specifically, in the emmetropic group, hyperopic defocus increased significantly at 60 min compared to baseline (estimate = 0.51, *p* = 0.006). In the myopic group, significant increases in hyperopic defocus were detected at 50 min (estimate = 0.57, *p* = 0.002) and 60 min (estimate = 0.70, *p* < 0.001). These findings suggest that prolonged near viewing of an electronic device induces a gradual hyperopic shift, with significant increases at later time points, reinforcing the time‐dependent nature of accommodative changes in both emmetropic and myopic children.

**FIGURE 7:**
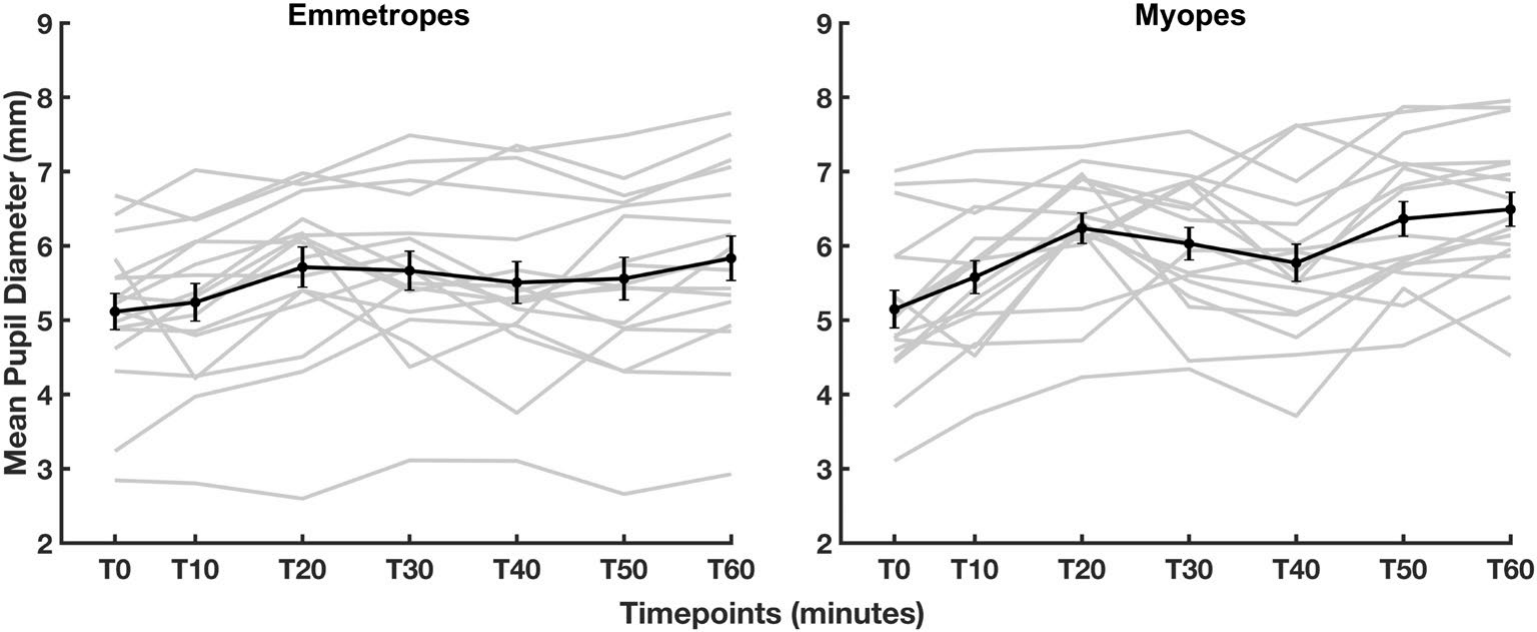
Mean pupil diameter (mm) as a function of time for all children. The mean pupil diameter for 17 children in each refractive group is represented by the black solid line and symbols for both emmetropic (left panel) and myopic (right panel) groups. Individual data points for each child are displayed in grey lines. Error bars indicate ±1 standard error of the mean. T0–T60 indicates the elapsed time in minutes.

Interestingly, both emmetropic and myopic children demonstrated a gradual, yet significant (*p* = 0.04) increase in pupil diameter over 60 min of sustained visual demand. Specifically, mean pupil diameter increased from 5.12 to 5.83 mm in emmetropic children and from 5.15 to 6.49 mm in myopic children. An LME model showed no significant difference between refractive groups (*p* = 0.78), indicating similar pupil responses (Figure 7).

**FIGURE 8:**
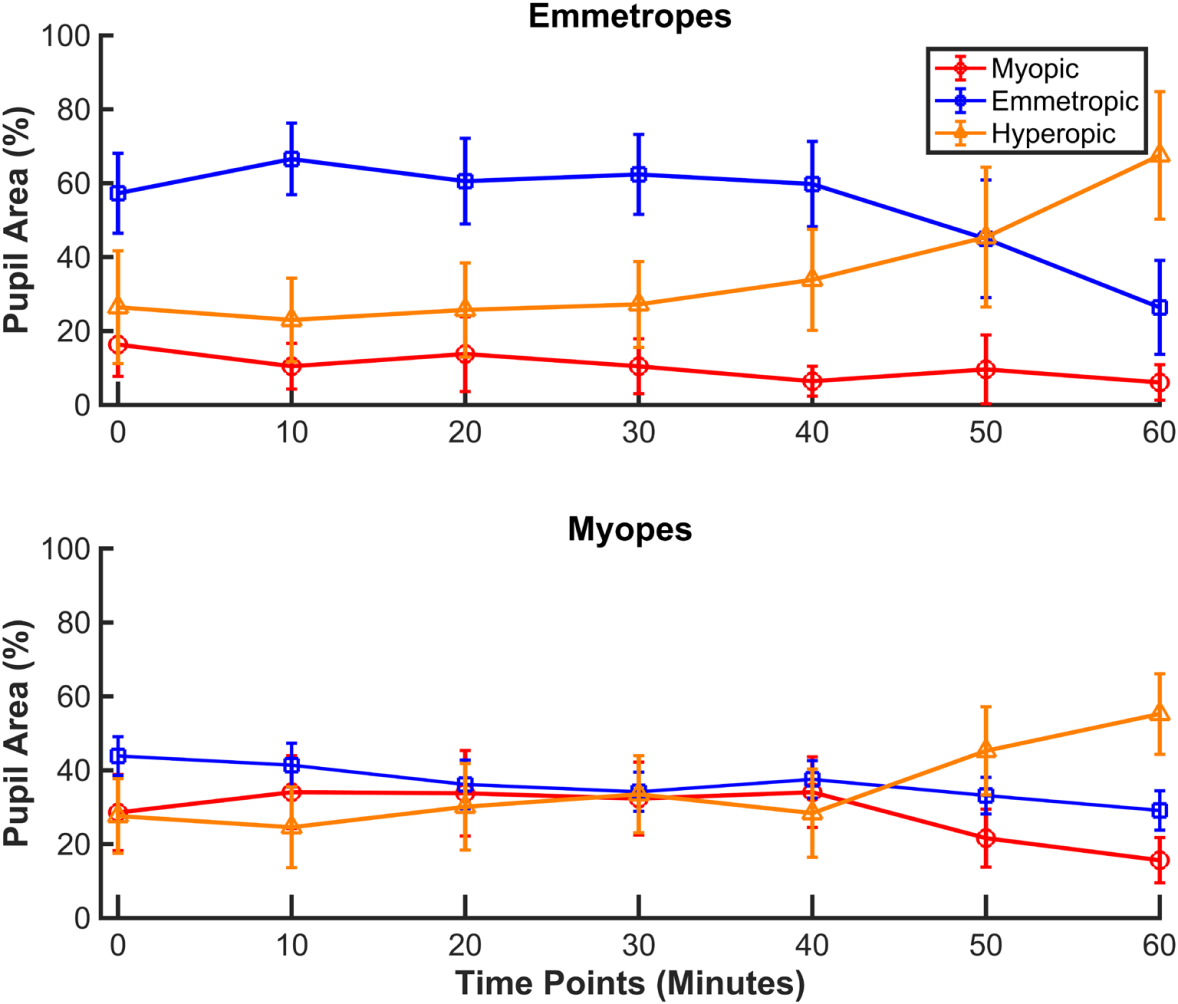
Line graphs illustrating the percentages of myopically (red), emmetropically (blue) and hyperopically (orange) defocused light on the retina across the full pupil during 60 min of electronic device exposure at a distance of 20 cm. The top panel present data for emmetropic children, while the bottom panel display data for myopic children. Data are shown for each time point from 0 to 60 min. Error bars indicate the 95% confidence interval.

One of the reported^40^ primary goals of DF optics is to counteract the chronic exposure of hyperopic defocus resulting from accommodative lag while also introducing controlled myopic defocus as a stimulus to slow axial eye growth. To evaluate data with this in mind, we examined how much light reaching the retina was either hyperopically or myopically defocused, considering the entire natural pupil. This assessment allows an understanding of the focused and defocused light present during extended near tasks. Both emmetropic and myopic groups exhibited an increase in the average proportion of hyperopically defocused light on the retina over time (Figure 8).

Specifically, for emmetropic children, the hyperopically defocused light (mean ± SE) increased from about 26 ± 15.26% at T0 to 45 ± 18.93% and 67 ± 17.25% by 50 and 60 min, respectively. For myopic children wearing DF CLs, the hyperopically defocused light increased from about 27 ± 10.11% at T0 to 45 ± 11.93% and 55 ± 10.90% by 50 and 60 min, respectively. Interestingly, while the amount of hyperopically defocused light increased for both groups, the DF CLs also demonstrated their ability to maintain myopic defocus throughout the viewing session. Specifically, the proportion of myopically defocused light was largely maintained, starting at 28.53 ± 10.31% at baseline and remaining steady at 22 ± 7.81% and 16 ± 6.14% at 50 and 60 min, respectively (Figure 8). A linear mixed model revealed no significant effects of group (myopes vs. emmetropes), time (T0–T60) or their interaction on hyperopically or myopically defocused light.

## DISCUSSION

This study investigated the accommodative behaviour and retinal defocus in children during a 60‐min session of sustained electronic device use at a near distance, specifically comparing the responses of uncorrected emmetropic children and myopic children wearing a DF CL. Previous studies have investigated the accommodative behaviour in eyes wearing multifocal or DF CLs either immediately^12^, ^34^, ^41^, ^42^, ^43^, ^44^, ^45^, ^46^ or during short‐term (<1 min)^47^, ^48^, ^49^ visual tasks. The present study extends this understanding by examining accommodative behaviour and retinal defocus changes after sustained near work for 60 min. Specifically, the current study used two approaches to quantify retinal defocus: First, by measuring the dioptric magnitude of induced hyperopic or myopic defocus for each zone of the DF CLs and second, by quantifying the proportion of light reaching the retina that was emmetropically, hyperopically or myopically defocused across the full pupil. The first approach highlights the dioptric impact of each zonal eye + lens area, whereas the second describes the distribution of defocus throughout the entire pupil, common to natural viewing.

The results align with previously published results indicating a promising ability of DF CLs to slow myopic eye growth and do so even under the potential negative effects of extended near tasks and the associated accommodative lags often reported.^11^, ^30^, ^50^, ^51^ It was observed that although accommodative lag did increase over the 60‐min near viewing period, in myopic children DF lenses still generally induced some myopic defocus throughout the period of electronic device exposure. However, due to the expected accommodated lag, there was a gradual decline in the magnitude of myopic defocus over time.

However, there remains an opportunity to explore further individualised accommodative responses and consider alternative lens designs that could sustain higher levels of myopic defocus (e.g., similar to those measured at baseline in the current study) during longer viewing periods. The current study, however, did utilise a potentially extreme scenario to represent the near work conditions common to today's children by having participants view at 20 cm continuously for 1 h while positioned on a chin rest. Specifically, although children may use devices for 4–6 h per day (for ages 8–12 years) or up to 8 h per day (for teenagers),^6^ viewing distances are often farther than 20 cm,^52^ and even if that is indeed the case, device use is often not continuous and typically only for increments of about 3.7 min at a time.^53^ Future work capturing task‐dependent viewing distance and behaviour will help provide context to the current results and may further drive clinical decision‐making.

In comparing the current results to previous studies,^12^, ^34^, ^54^ a similar consistent pattern in the accommodative responses of myopic children was observed, whether wearing single vision lenses or MiSight 1‐day lenses. These children typically exhibit low levels of myopic defocus while fixating on distant targets, transitioning to hyperopic defocus as the target becomes closer.^12^, ^34^ Although this study did not measure the prolonged accommodative behaviour of myopes directly while wearing single vision CLs for up to 1 h of continuous near viewing, existing literature^12^, ^34^, ^51^, ^55^ suggests responses are likely to be similar to those observed in the current study, with an increased lag over time.

As typical to study design, this study has several limitations. Participants were not adapted DF CL wearers. Although previous research has shown that accommodative behaviour in children adapts quickly, typically within a few minutes of lens wear,^56^, ^57^, ^58^ the prolonged accommodative response of adapted children represents a future opportunity for improved understanding. The experimental set‐up utilised an open‐view aberrometer and a fixed 20 cm viewing distance for 1 h, representing a controlled, highly demanding scenario of near work. In real‐world conditions, device use in children typically involves variable distances (e.g., 25–40 cm), intermittent posture shifts and micro‐breaks.^59^ For example, reading a book, playing video games or engaging in interactive educational apps may demand different levels of cognitive and visual processing compared to passively watching a movie.^60^, ^61^, ^62^, ^63^, ^64^ Ideally, at some point, future studies with advanced technology will be able to capture real‐world, naturalistic data. However, until that technology exists, the current study valuably benchmarks a potential ‘highly demanding’ scenario in a controlled way and importantly still finds the myopia control lenses to introduce levels of myopic defocus effectively. Although lens decentration was included for myopic eyes wearing MiSight CLs, no decentration was assumed for the equivalent zone geometry analysis for emmetropic eyes. This assumption may impact the results slightly, but given the slightly aspheric nature of uncorrected eyes, it is not expected to be large. This study evaluated only the MiSight 1‐day DF lens, a representative myopia control design, characterised by its specific alternating distance and near zone configuration. Although theoretical differences could occur, similar trends from other optical designs are anticipated given the reported similarity in short‐term^41^, ^65^ measures of accommodative responses with myopia control lenses. While the sample size (*n* = 34) was sufficient for detecting significant effects in this detailed optical analysis, as with any study, some caution is suggested when generalising findings across the full diversity of populations or age groups. The age range of children in this current study spanned 7–17 years, yet the mean sample age skewed towards adolescence. Of course, younger children (<10 years of age) may exhibit greater variability in near‐working distances^52^ and accommodative responses,^34^ but the present sample size was insufficient to explore these fully. Further work should examine age‐related differences in the accommodative responses. Importantly, the age range of the lens users was also skewed to align closely with the sample distribution, somewhat minimising this concern.

In summary, this study elucidated the accommodative behaviour and retinal defocus in children wearing DF CLs during 1 h of sustained near work at 20 cm. The findings demonstrate that DF CLs introduced myopic defocus even during sustained near viewing at a very close working distance, supporting their reported efficacy in myopia control. These results also reveal a progressive relative increase in hyperopic defocus over 50–60 min of sustained device viewing at near, indicating some potential for further optimised strategies which may possibly enhance myopia management in clinical practice. Specifically, we recommend encouraging children to: (1) maintain viewing distances >20 cm during sustained near viewing (e.g., digital device use) to reduce accommodative demand and subsequent hyperopic defocus and (2) incorporate near work breaks (e.g., at least every 30–40 min) to allow accommodative relaxation and not stay focused on the near work. We believe these patient‐ and parent‐education measures may have the potential to complement DF lens myopia control treatment.

## AUTHOR CONTRIBUTIONS

**Neeraj K. Singh:** Conceptualization (lead); data curation (equal); formal analysis (lead); investigation (lead); methodology (lead); project administration (equal); resources (equal); software (equal); validation (equal); visualization (equal); writing – original draft (lead); writing – review and editing (equal). **Pete Kollbaum:** Conceptualization (equal); data curation (equal); formal analysis (equal); investigation (equal); methodology (equal); project administration (equal); resources (equal); software (equal); supervision (equal); validation (equal); visualization (equal); writing – original draft (equal); writing – review and editing (equal).

## FUNDING INFORMATION

None.

## CONFLICT OF INTEREST STATEMENT

PK serves as a consultant for EssilorLuxottica, and has research funding from EssilorLuxottica, Alcon, CooperVision, Hoya, SightGlass Vision, and the National Eye Institute (NEI).
